# Etiologies of influenza-like illness and severe acute respiratory infections in Tanzania, 2017–2019

**DOI:** 10.1371/journal.pgph.0000906

**Published:** 2023-02-09

**Authors:** Maria Ezekiely Kelly, Radhika Gharpure, Sabrina Shivji, Miriam Matonya, Solomon Moshi, Ambele Mwafulango, Vumilia Mwalongo, Janneth Mghamba, Azma Simba, S. Arunmozhi Balajee, Wangeci Gatei, Marcelina Mponela, Grace Saguti, Toni Whistler, Nyambura Moremi, Vida Mmbaga

**Affiliations:** 1 Ministry of Health, Dar es Salaam, Tanzania; 2 U.S. Centers for Disease Control and Prevention, Atlanta, Georgia, United States of America; 3 U.S. Centers for Disease Control and Prevention, Dar es Salaam, Tanzania; 4 World Health Organization, Dar es Salaam, Tanzania; PLOS: Public Library of Science, UNITED STATES

## Abstract

In 2016, Tanzania expanded sentinel surveillance for influenza-like illness (ILI) and severe acute respiratory infection (SARI) to include testing for non-influenza respiratory viruses (NIRVs) and additional respiratory pathogens at 9 sentinel sites. During 2017–2019, respiratory specimens from 2730 cases underwent expanded testing: 2475 specimens (90.7%) were tested using a U.S. Centers for Disease Control and Prevention (CDC)-developed assay covering 7 NIRVs (respiratory syncytial virus [RSV], rhinovirus, adenovirus, human metapneumovirus, parainfluenza virus 1, 2, and 3) and influenza A and B viruses. Additionally, 255 specimens (9.3%) were tested using the Fast-Track Diagnostics Respiratory Pathogens 33 (FTD-33) kit which covered the mentioned viruses and additional viral, bacterial, and fungal pathogens. Influenza viruses were identified in 7.5% of all specimens; however, use of the CDC assay and FTD-33 kit increased the number of specimens with a pathogen identified to 61.8% and 91.5%, respectively. Among the 9 common viruses between the CDC assay and FTD-33 kit, the most identified pathogens were RSV (22.9%), rhinovirus (21.8%), and adenovirus (14.0%); multi-pathogen co-detections were common. Odds of hospitalization (SARI vs. ILI) varied by sex, age, geographic zone, year of diagnosis, and pathogen identified; hospitalized illnesses were most common among children under the age of 5 years. The greatest number of specimens were submitted for testing during December–April, coinciding with rainy seasons in Tanzania, and several viral pathogens demonstrated seasonal variation (RSV, human metapneumovirus, influenza A and B, and parainfluenza viruses). This study demonstrates that expanding an existing influenza platform to include additional respiratory pathogens can provide valuable insight into the etiology, incidence, severity, and geographic/temporal patterns of respiratory illness. Continued respiratory surveillance in Tanzania, and globally, can provide valuable data, particularly in the context of emerging respiratory pathogens such as SARS-CoV-2, and guide public health interventions to reduce the burden of respiratory illnesses.

## Introduction

At the global level, influenza surveillance is crucial to monitoring trends of seasonal circulation, guiding strain selection for vaccine composition, detecting the emergence of pandemic-potential influenza viruses, and monitoring their impact and spread. At the country level, the data can be used to inform and promote prevention strategies and provide information for policymaking and public health interventions such as vaccine implementation. In 2008, the Tanzanian Ministry of Health established influenza sentinel surveillance at 5 hospitals across the country and reported on the first 30 months of data [1]. The surveillance system covered both influenza-like illness (ILI) outpatient consultation and severe acute respiratory infection (SARI) hospitalizations. Since 2009, the National Influenza Center in Dar es Salaam has regularly uploaded data to the World Health Organization (WHO) FluNet system, providing valuable information on East African influenza activity and contributing to global biannual influenza vaccine strain recommendations. Further, the sentinel sites share data weekly with the Ministry of Health through the country’s Integrated Disease and Surveillance Response system. These surveillance data have been used thus far to characterize the national burden of influenza and in the future, the data could be used to develop a national action plan for influenza vaccine introduction in the country.

Kishamawe et al. [2] evaluated patterns and causes of respiratory-related deaths in hospitals in Tanzania over a 10-year period (2006–2015) and demonstrated that respiratory diseases, predominantly acute and chronic respiratory infections, accounted for a substantial proportion (12.92%) of all hospital deaths in Tanzania, with nearly one third of those deaths occurring in children under 5 years of age. This 2019 publication emphasized the importance of strengthening laboratory capacity to identify the causative agents of respiratory diseases. In many countries undertaking respiratory disease surveillance, influenza virus is the pathogen most frequently tested for and has received the most attention; however, an analysis of influenza sentinel surveillance from 15 countries in Africa indicated that during 2006–2010, only 22% of ILI and 10% of SARI cases were positive for influenza virus [3]. Similarly, from 2012 to 2017, country-specific influenza sentinel surveillance data from Africa showed that between 15–30% of ILI and 3–7% of SARI cases were positive for influenza [4–7]. Given the high proportion of influenza-negative specimens, other respiratory pathogens play a significant role in respiratory disease burden, highlighting the need for expanded laboratory testing for non-influenza respiratory viruses (NIRVs) within ILI/SARI surveillance networks. In 2016, as a component of the Global Health Security Agenda, the existing influenza sentinel surveillance for ILI/SARI in Tanzania was extended from 5 sites to 9 sites and testing expanded to include 7 NIRVs and additional respiratory pathogens. This study summarizes expanded ILI/SARI surveillance findings from 2017 through 2019 to address knowledge gaps in etiology, incidence, severity, and geographic/temporal patterns of ILI/SARI in Tanzania.

## Materials and methods

### Surveillance sites and case enrollment

**Fig 1:**
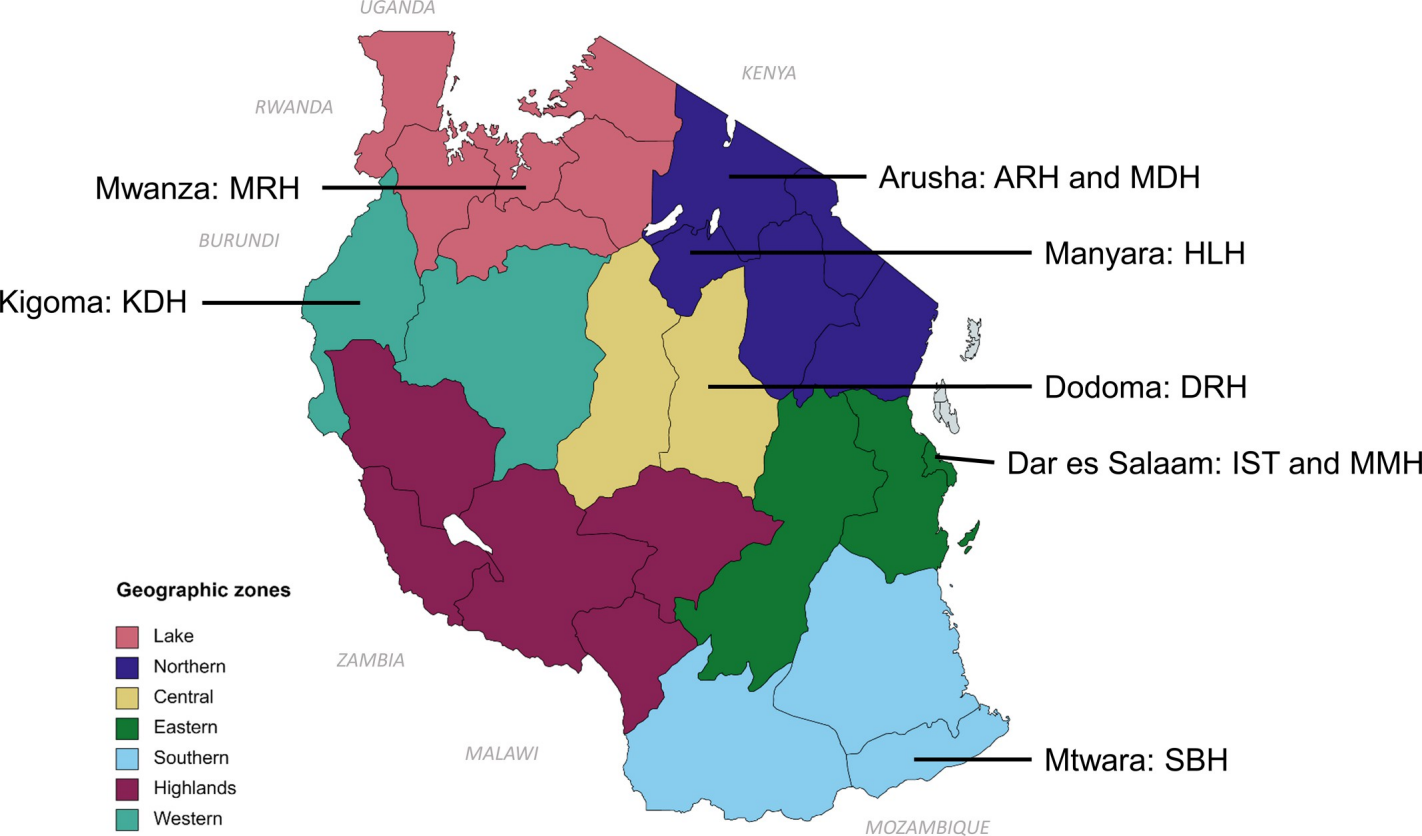
Influenza -like illness (ILI) and severe acute respiratory infection (SARI) sentinel surveillance sites in Tanzania, by region and geographic zone. The map shows the 21 regions (official administrative areas) and 7 geographic zones of mainland Tanzania. The 9 sentinel sites for ILI/SARI surveillance are shown in their respective regions: Kigoma: Kibondo District Hospital (KHD); Mwanza: Mwanza Regional Hospital (MRH); Arusha: Arusha Regional Hospital (ARH) and Meru District Hospital (MDH); Manyara: Haydom Lutheran Hospital (HLH); Dodoma: Dodoma Regional Hospital (DRH); Dar es Salaam: Mwananyamala Municipal Hospital (MMH) and International School of Tanganyika Clinic (IST)*; Mtwara: St. Benedict Referral Hospital (SBH). Map created with MapChart (openly available source): https://www.mapchart.net/. Republished from MapChart under a CC BY license with permission (S1 Text), original copyright 2022. *IST only collected ILI data.

This was a subset study of Tanzania’s influenza sentinel surveillance [1] and no additional patient characteristics were considered. As such, patient enrollment followed the routine national protocol for influenza sentinel surveillance as part of the WHO Global Influenza Surveillance Network. Specimens for expanded respiratory testing were selected from all nine ILI/SARI sentinel surveillance sites (Fig 1). This included 8 hospitals, both regional and district, and 1 clinic, International School of Tanganyika Clinic, which only collected ILI data. The ILI/SARI patient enrollment and sampling strategy used WHO case definitions for ILI and SARI [8] and included all patients meeting the daily sample size for sentinel surveillance as previously described (1). The ILI case definition required an acute respiratory infection with documented fever (axillary temperature ≥ 38°C) and cough with onset within the last 10 days. The SARI definition required history of fever or measured fever at presentation, in addition to cough, both with onset within 10 days of presentation; additionally, illness required hospitalization.

### Specimen collection and transport

Both a nasopharyngeal and an oropharyngeal swab were collected from all enrolled ILI/SARI cases. Both swabs were placed into a single tube of viral transport medium and immediately refrigerated at 4°C–8°C. Specimens from hospitals outside of Dar es Salaam were triple-packaged and transported in cooler boxes to the National Health Laboratory Quality Assurance and Training Centre by courier service, all others were hand carried to the laboratory. All specimens were immediately stored at −80°C.

### Laboratory testing methods

Two multi-pathogen testing modalities were implemented to conduct expanded respiratory pathogen testing during this surveillance period; the first was a combination of real-time RT-PCR singleplex assays developed by the US Centers for Disease Control and Prevention (US CDC) and the second was the Fast Track Diagnostics Respiratory Pathogens 33 (FTD-33) multiplex kit, available during 2018–2019. Specimen selection for expanded respiratory testing was based on a few criteria: 50% of all influenza-negative specimens were randomly selected each month and tested using the CDC RT-PCR singleplex assays; an additional 10–20 samples were randomly selected each quarter from total surveillance samples received (regardless of influenza positivity) and tested using the FTD-33 kit.

### CDC expanded respiratory virus real-time RT-PCR singleplex assays

Most specimens were tested using a 9-assay respiratory RNA virus panel of singleplex reverse transcription real-time polymerase chain reactions (RT-qPCRs) developed by the Division of Viral Diseases at the National Center for Immunization and Respiratory Diseases, US CDC. The assays were for: influenza A and influenza B viruses, respiratory syncytial virus (RSV; both A and B subtypes); human metapneumovirus (hMPV; both A and B subtypes); human parainfluenza viruses 1 (PIV1), 2 (PIV2), and 3 (PIV3); human rhinovirus; and human adenovirus. In the testing algorithm, all influenza A virus-positive samples were further characterized using the RT-qPCR influenza A virus (H3, H1pdm09) subtyping panel (RUO). All reagents for testing were obtained from the International Reagent Resource (IRR; https://www.internationalreagentresource.org) at no cost.

Briefly, RNA was extracted from 140μl of specimen using a QIAamp Viral RNA Mini Kit (Qiagen, Germany) according to the manufacturer’s instructions. Amplification was performed using the AgPath-ID One-Step RT-PCR Reagents (Applied Biosystems, Carlsbad, CA). Each reaction mix consisted of 5 μl nucleic acid extract and 20 μl master mix (0.5 μl SuperScript III One-Step RT-PCR System with Platinum Taq DNA Polymerase (Life Technologies), 12.5 μl 2X reaction mix, 0.5 μl RNaseOUT Recombinant Ribonuclease Inhibitor (Invitrogen, USA), 0.5μl of each primer (20 μM) and 0.5μl of probe (6 μM), and 5μl RNase-free water). Assays were run on the Applied Biosystems 7500 Real-Time PCR instrument (Thermo Fisher Scientific) with cycling conditions of: 50°C for 30 minutes (reverse transcription), 95°C for 2 minutes (inactivation/denaturation), and 45 cycles of 95°C for 15 seconds and 62°C for 30 seconds (amplification). Appropriate negative and positive control specimens were run alongside each reaction. When all controls met the stated requirements, an assay was considered positive if it had a well-defined curve that crossed the cycle threshold within 40 cycles.

### Fast track diagnostics respiratory pathogens (FTD-33) multiplex kit

The FTD-33 kit detects the following respiratory viruses, bacteria, and fungi: influenza A, influenza A subtype A(H1N1)pdm09, influenza B, and influenza C; PIV1, PIV2, PIV3, and PIV4; coronaviruses NL63, 229E, OC43, and HKU1; hMPV A and B; rhinovirus, RSV A and B; adenovirus; enterovirus; parechovirus; bocavirus; and *Pneumocystis jirovecii*, *Mycoplasma pneumoniae*, *Chlamydia pneumoniae*, *Streptococcus pneumoniae*, *Haemophilus influenzae*, *Haemophilus influenzae* type B, *Staphylococcus aureus*, *Moraxella catarrhalis*, *Bordetella* species (excluding *B*. *parapertussis*), *Klebsiella pneumoniae*, *Legionella* species, and *Salmonella* species. During 2018–2019, approximately 10% of ILI/SARI specimens were randomly selected for testing using the FTD-33 kit.

A total nucleic extract was made from 140μl of specimen using the QIAamp Viral RNA Mini kit (Qiagen, Hilden, Germany) according to manufacturer’s instructions for use. Each extract was tested by the FTD method using 10μl of extract. The eight multiplex RT-qPCR reactions were set up following the manufacturer’s instructions.

An extraction control, no-template control and positive control plasmid pools were included for testing of the eight multiplex reaction mixes and the RNase P reaction mix. All assays were amplified using the Applied Biosystems 7500 Real-Time PCR Instrument (Thermo Fisher Scientific) with the following cycling conditions: 42°C for 15 minutes, 94°C for 3 minutes, 40 cycles of 94°C for 8 seconds, and 60°C for 34 seconds.

### Statistical analysis

Descriptive statistics were used to summarize the continuous and discrete variables. Categorical variables were presented as numbers or percentages, and Pearson’s Chi-square tests were used to compare groups. Continuous variables were presented as median with interquartile range (IQR). Logistic regression was used to assess the odds of hospitalization. Percent positivity (defined as number of specimens with a positive result among all specimens tested) was aggregated by month for the nine pathogens to evaluate monthly and seasonal distribution. All analyses were performed using SAS statistical software (version 9.4; SAS Institute, Cary, NC).

### Ethics statement

The Tanzanian Ministry of Health and Social Welfare determined that influenza sentinel surveillance was considered routine public health surveillance and therefore formal ethical review was not required. Verbal consent was obtained from all patients before questionnaires were administered and specimens were collected. The ILI and SARI surveillance activities in Tanzania were reviewed by U.S. CDC and conducted consistent with applicable federal law and U.S. CDC policy.

Additional information regarding the ethical, cultural, and scientific considerations specific to this surveillance activity is included in the Supporting Information (S2 Text).

## Results

During 2017–2019, a total of 5448 specimens were submitted for influenza sentinel surveillance laboratory testing. Of these, 2730 (50.1%) specimens underwent expanded respiratory pathogen testing. Of these, 2475 (90.7%) specimens were tested with the CDC singleplex assays, and 255 specimens (9.3%) were tested using the FTD-33 kit. Patient demographics and identification of the nine common viruses were comparable overall across the two assays (S1 Table), allowing for results to be combined.

Of the 2730 patients whose specimens were tested, 920 (33.7%) cases were classified as ILI and 1810 (66.3%) as SARI. Median age was 2 years (IQR: 0.75–14) overall; among persons with ILI, median age was 7 years (IQR: 1–33) and among persons with SARI, median age was 1 year (IQR: 0.58–3). Odds of SARI hospitalization (defined as SARI vs. ILI) varied by sex, age, geographic zone, and year of diagnosis (Table 1). Specifically, SARI cases were more common among males, children under the age of 5 years, in the Eastern, Central, and Western zones, and in 2017 compared with 2018–2019.

**Table 1:** Patient demographics, geographic zone, year, and diagnostic assay used for respiratory pathogen detection among ILI/SARI cases—Tanzania, 2017–2019.

	Total	ILI	SARI	p-value^a^
	(n = 2730)	(n = 920)	(n = 1810)	
	*N*	*n (%)*	*n (%)*	
**Sex**				
Female	1247	498 (54.1)	749 (41.4)	
Male	1483	422 (45.9)	1061 (58.6)	<0.0001
**Age group** (years)				
<1	880	168 (18.2)	712 (39.3)	
1 –<5	982	250 (27.2)	732 (40.4)	
5 –<18	220	111 (12.1)	109 (6.0)	
18 –<65	559	360 (39.1)	199 (11.0)	
≥65	89	31 (3.4)	58 (3.2)	<0.0001
**Geographic zone** ^b^				
Northern	834	338 (36.7)	496 (27.4)	
Eastern	805	237 (25.8)	568 (31.4)	
Central	125	12 (1.3)	113 (6.2)	
Southern	260	95 (10.3)	164 (9.1)	
Western	453	87 (9.5)	366 (20.2)	
Lake	253	151 (16.4)	102 (5.6)	<0.0001
**Year**				
2017 (n = 2093)^c^	483	119 (12.9)	364 (20.1)	
2018 (n = 1642)^c^	973	311 (33.8)	662 (36.6)	
2019 (n = 1713)^c^	1274	490 (53.3)	784 (43.3)	<0.0001
**Diagnostic assay**				
CDC	2475	821 (89.2)	1654 (91.4)	
FTD-33	255	99 (10.8)	156 (8.6)	0.07

Abbreviations: ILI, influenza-like illness; SARI, severe acute respiratory infection; CDC, U.S. Centers for Disease Control and Prevention singleplex assays; FTD-33, Fast-Track Diagnostics respiratory panel multiplex kit.

^a^p-value for chi-squared test comparing ILI and SARI cases.

^b^Northern zone includes Arusha Regional Hospital (Arusha), Meru District Hospital (Arusha), and Haydom Lutheran Hospital (Manyara); Eastern includes International School of Tanganyika Clinic (Dar es Salaam) and Mwananyamala Municipal Hospital (Dar es Salaam); Central includes Dodoma Regional Hospital (Dodoma); Southern includes St. Benedict Referral Hospital (Mtwara); Western includes Kibondo District Hospital (Kigoma); and Lake includes Mwanza Regional Hospital (Mwanza).

^c^Numbers in brackets show the total SARI/ILI specimens collected each year.

**Fig 2:**
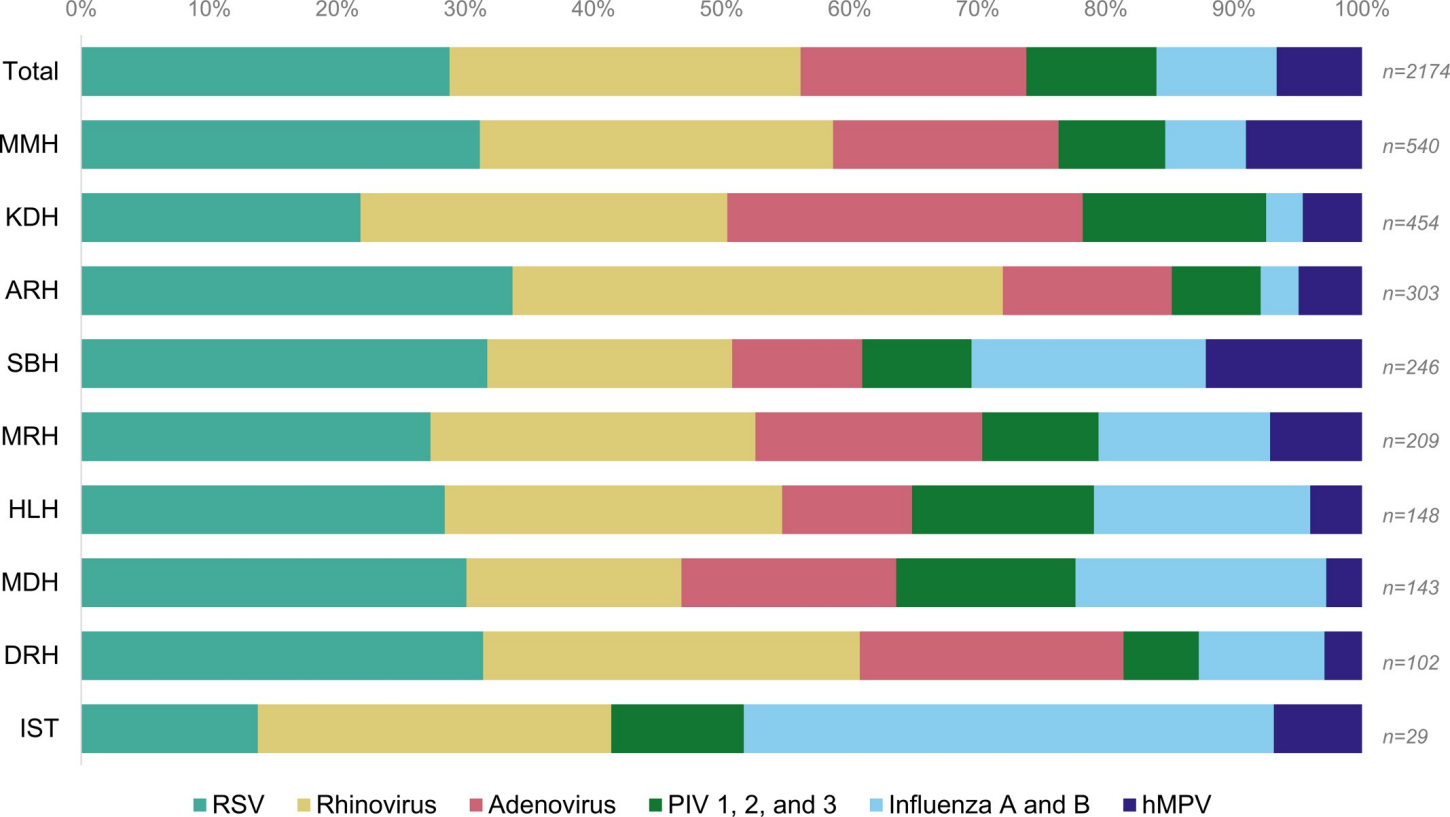
Respiratory virus detection, in total and by ILI/SARI surveillance site—Tanzania, 2017–2019. Abbreviations: ILI, influenza-like illness; SARI, severe acute respiratory infection; MMH, Mwananyamala Municipal Hospital (Dar es Salaam); KDH, Kibondo District Hospital (Kigoma); ARH, Arusha Regional Hospital (Arusha); SBH, St. Benedict Referral Hospital (Mtwara); MRH, Mwanza Regional Hospital (Mwanza); HLH; Haydom Lutheran Hospital (Manyara); MDH, Meru District Hospital (Arusha); DRH, Dodoma Regional Hospital (Dodoma); IST, International School of Tanganyika Clinic* (Dar es Salaam); n, number of specimens tested; RSV, respiratory syncytial virus; hMPV, human metapneumovirus; PIV, parainfluenza virus (1, 2, and 3). *IST only collected ILI data.

The most identified viral pathogens were RSV (22.9%), rhinovirus (21.8%), and adenovirus (14.0%) (Table 2). These three pathogens were most identified among children <1 year old and 1–<5 years old. Among all age groups, 1044 specimens (38.2%) had none of the nine viral pathogens identified; the proportion of cases without any pathogen identified increased with age and was greatest among individuals ≥65 years old (56.2%). We identified co-detection (detection of >1 of the nine common pathogens) in 420 (15.4%) cases; pathogens most identified in co-detections were RSV, rhinovirus, and adenovirus (S2 Table). Distribution of pathogens identified varied slightly by geographic location (Fig 2).

**Table 2:** Viral respiratory pathogens detected, by age group, among specimens tested from ILI/SARI cases, Tanzania, 2017–2019.

Pathogen detected^a^	Total	Age group (years)				
		n (%)				
	(N = 2730)	<1	1 –<5	5 –<18	18 –<65	≥65
		(n = 880)	(n = 982)	(n = 220)	(n = 559)	(n = 89)
RSV	625 (22.9)	278 (31.6)	233 (23.7)	33 (15.0)	75 (13.4)	6 (6.7)
Rhinovirus	596 (21.8)	208 (23.6)	217 (22.1)	48 (21.8)	110 (19.7)	13 (14.6)
Adenovirus	383 (14.0)	146 (16.6)	166 (16.9)	14 (6.3)	49 (8.8)	8 (9.0)
hMPV	145 (5.3)	53 (6.0)	52 (5.3)	12 (5.5)	25 (4.5)	3 (3.4)
Influenza A virus^b^	131 (4.8)	32 (3.6)	45 (4.6)	18 (8.2)	32 (5.7)	4 (4.5)
PIV3	125 (4.6)	42 (4.8)	48 (4.9)	7 (3.2)	23 (4.1)	5 (5.6)
Influenza B virus	73 (2.7)	13 (1.5)	22 (2.2)	13 (5.9)	22 (3.9)	3 (3.4)
PIV1	62 (2.3)	16 (1.8)	25 (2.6)	6 (2.7)	13 (2.3)	2 (2.3)
PIV2	34 (1.3)	6 (0.7)	18 (1.8)	1 (0.5)	9 (1.6)	0 (0)
No pathogen identified^c^	1044 (38.2)	276 (31.4)	348 (35.4)	91 (41.4)	279 (49.9)	50 (56.2)

Abbreviations: ILI, influenza-like illness; SARI, severe acute respiratory infection; N/n, number of specimens tested; RSV, respiratory syncytial virus; hMPV, human metapneumovirus; PIV, parainfluenza virus (1, 2, and 3); CDC, U.S. Centers for Disease Control and Prevention singleplex assays; FTD-33, Fast-Track Diagnostics respiratory panel multiplex kit.

^a^ Sum of column percentages exceeds 100% as multiple viruses were detected in specimens from 420 patients.

^b^ Includes A/H3 (n = 78) and A/H1N1pdm (n = 53).

^c^Nine viruses included on both CDC and FTD-33 diagnostic assays; additional viral pathogens were tested using the FTD-33 kit on a subset of specimens and were not included in this analysis.

Upon inclusion of the additional 24 viral and bacterial pathogens tested in the FTD-33 kit only, the proportion of patients without any pathogen identified decreased to 9.4%; children ages 1–<5 years old, followed by adults 18–<65 years were the most common age groups without any pathogen identified for the FTD-33 assay (Table 3). Mixed viral and bacterial infections were common (52.9%); the most identified viruses in mixed co-detections were RSV, rhinovirus, and adenovirus, and the most identified bacteria were *Klebsiella pneumoniae*, *Streptococcus pneumoniae*, and *Moraxella catarrhalis*. Bacterial pathogens, including co-detections, were most identified in patients <1 year old and 1–<5 years old.

**Table 3:** Expanded bacterial and viral respiratory pathogen testing performed on subset of patient specimens using FTD-33 multiplex kit—Tanzania, 2018–2019^a^.

Variable	Pathogen detection						
	n (%)						
	Total	None	Single		Multiple		
			Virus^b^	Bacterium^c^	Viral^d^	Bacterial^e^	Mixed^f^
	(N = 255)	(n = 24)					
			(n = 21)	(n = 36)	(n = 2)	(n = 37)	(n = 135)
**Classification**							
ILI	99 (38.8)	11 (45.8)	10 (47.6)	13 (36.1)	0 (0)	9 (24.3)	56 (41.5)
SARI	156 (61.2)	13 (54.2)	11 (52.4)	23 (63.9)	2 (100.0)	28 (75.7)	79 (58.5)
**Age group** (years)							
<1	78 (30.6)	2 (8.3)	7 (33.3)	10 (27.8)	0 (0)	12 (32.4)	47 (34.8)
1 –<5	101 (39.6)	9 (37.5)	6 (28.6)	14 (38.9)	1 (50.0)	14 (37.8)	57 (42.2)
5 –<18	20 (7.8)	4 (16.7)	2 (9.5)	3 (8.3)	0 (0)	4 (10.8)	7 (5.2)
18 –<65	51 (20.0)	8 (33.3)	3 (14.3)	9 (25.0)	1 (50.0)	7 (18.9)	23 (17.0)
≥65	5 (2.0)	1 (4.2)	3 (14.3)	0 (0.0)	0 (0)	0 (0)	1 (0.7)

Abbreviations: N/n, number of specimens tested; FTD-33, Fast-Track Diagnostics multiplex kit; ILI, influenza-like illness; SARI, severe acute respiratory infection.

^a^ No specimens were tested using the FTD-33 kit in 2017.

^b^ Most commonly identified single viral pathogens were respiratory syncytial virus (n = 12); rhinovirus (n = 4); and human metapneumovirus (n = 2).

^c^ Most commonly identified single bacterial pathogens were *Klebsiella pneumoniae* (n = 14); *Bordetella pertussis* (n = 8); and *Streptococcus pneumoniae* (n = 5).

^d^ Viral co-detections identified were adenovirus and rhinovirus (n = 1) and rhinovirus and influenza C virus (n = 1).

^e^ Bacteria most identified in bacterial co-detections were *S*. *pneumoniae* (n = 22); *Moraxella catarrhalis* (n = 20); and *K*. *pneumoniae* (n = 15).

^f^ Viruses most identified in mixed viral/bacterial co-detections were respiratory syncytial virus (n = 56); rhinovirus (n = 40); and adenovirus (n = 34). Bacteria most identified in mixed viral/bacterial co-detections were *K*. *pneumoniae* (n = 70); *S*. *pneumoniae* (n = 62); and *M*. *catarrhalis* (n = 59).

We assessed odds of SARI hospitalization against outpatient ILI among the 1266 patients with a single viral pathogen identified, as compared to those without any pathogen identified (Table 4). After adjusting for age, sex, and site, hospitalization was less common among patients whose specimens yielded rhinovirus (odds ratio [OR]: 0.69; 95% confidence interval [CI]: 0.52–0.91) and parainfluenza 3 virus (OR: 0.52; 95% CI: 0.29–0.92). Patients in the <1 year and 1–<5 years age groups had the greatest number of hospitalizations from all viruses (S3 Table).

**Table 4:** Odds of hospitalization (severe acute respiratory infection vs. influenza-like illness), by viral respiratory pathogen, among enrolled cases with a single viral pathogen detected^a^, Tanzania 2017–2019.

Pathogen	Total	ILI	SARI	Odds of hospitalization^b^	Odds of hospitalization, adjusted^c^
	(n = 2310)	(n = 782)	(n = 1528)	*OR (95% CI)*	*aOR (95% CI)*
	*N*	*n (%)*	*n (%)*		
RSV	409	122 (29.8)	287 (70.2)	1.14 (0.89–1.46)	0.93 (0.70–1.24)
Rhinovirus	388	140 (36.1)	248 (63.9)	0.86 (0.67–1.10)	**0.69 (0.52–0.91)**
Adenovirus	163	51 (31.3)	112 (68.7)	1.06 (0.74–1.51)	0.78 (0.52–1.16)
Influenza A virus	86	38 (44.2)	48 (55.8)	**0.61 (0.39–0.95)**	0.98 (0.58–1.68)
hMPV	69	21 (30.4)	48 (69.6)	1.10 (0.65–1.87)	1.06 (0.59–1.90)
PIV3	66	31 (47.0)	35 (53.0)	**0.55 (0.33–0.90)**	**0.52 (0.29–0.92)**
Influenza B virus	48	27 (56.3)	21 (43.8)	**0.38 (0.21–0.67)**	0.84 (0.43–1.66)
PIV1	26	7 (26.9)	19 (73.1)	1.31 (0.55–3.15)	1.70 (0.58–4.96)
PIV2	11	5 (45.5)	6 (54.6)	0.58 (0.18–1.91)	0.65 (0.19–2.29)
No virus identified^d^	1044	340 (32.6)	704 (67.4)	Ref.	Ref.

Abbreviations: N/n, number of enrolled cases; OR, odds ratio; aOR, adjusted odds ratio; CI, confidence interval; RSV, respiratory syncytial virus; hMPV, human metapneumovirus; PIV, parainfluenza virus (1, 2, and 3); Ref., reference group.

^a^ Patients with multiple viruses detected (n = 420) were not included in this analysis.

^b^ Odds of SARI vs. ILI, as a binary outcome.

^c^ Adjusted for age, sex, and hospital site.

^d^ Among nine viruses included on both CDC and FTD-33 diagnostic assays; additional viral pathogens were tested using the FTD-33 kit on a subset of specimens and were not included in this analysis.

**Fig 3:**
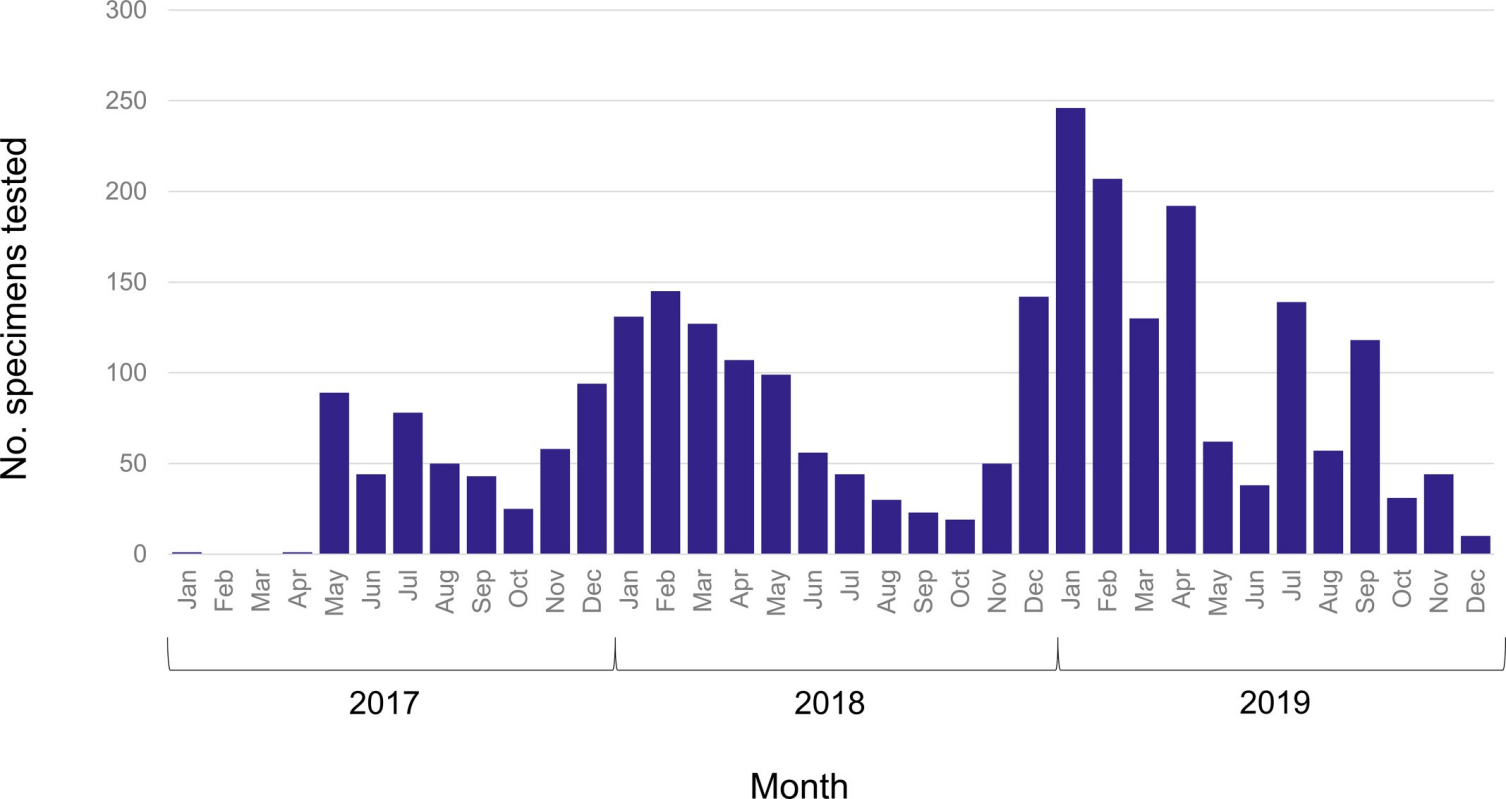
Number of specimens tested for viral respiratory pathogens by month, Tanzania 2017–2019.

**Fig 4:**
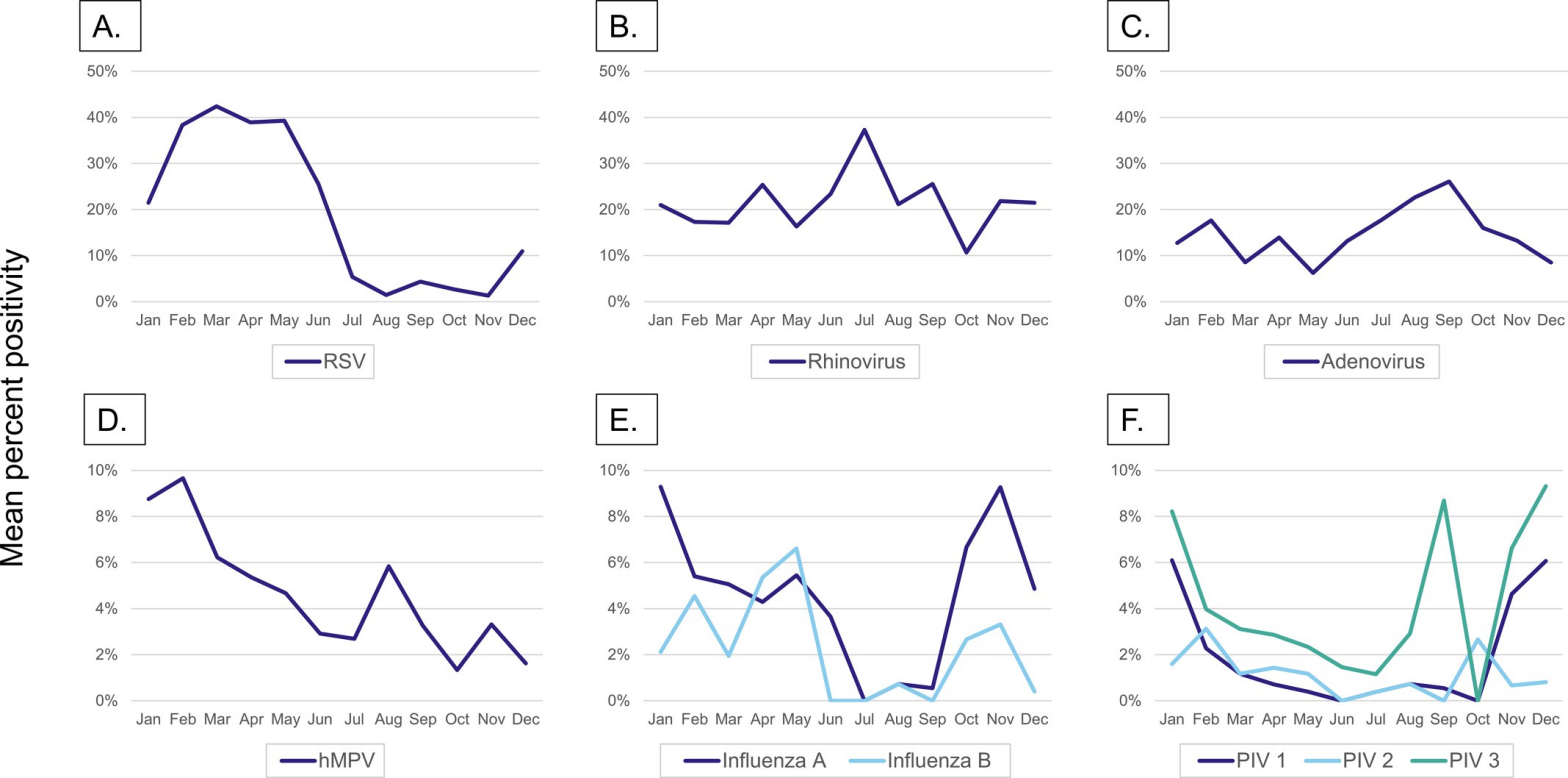
Monthly mean percent positivity of viral respiratory pathogens, Tanzania 2017–2019. Percent positivity by month for respiratory syncytial virus (A), rhinovirus (B), adenovirus (C), human metapneumovirus (hMPV) (D), influenza A and B viruses (E), and parainfluenza viruses (PIV1, PIV2, and PIV3) (F).

We observed a seasonal pattern in the number of specimens submitted by month during 2017–2019 (Fig 3); the greatest number were submitted during the months of December–April. We observed a seasonal pattern in RSV positivity, with greatest virus detection during January–May across the study period (Fig 4). Additionally, hMPV, influenza A and B viruses, and PIVs also demonstrated some evidence of seasonality, with a peak during January–May for hMPV, two peaks during April–May and October–December for influenza viruses, and peaks in November–January for PIVs. No apparent seasonality was observed for rhinovirus or adenovirus.

## Discussion

Influenza sentinel surveillance began in Tanzania in 2008 and the first report of data from this system [1] showed that 8.0% of ILI and SARI cases were positive for influenza virus during May 2008–November 2010. This is highly comparable to the 7.5% influenza positivity (4.8% influenza A; 2.7% influenza B) for the 3 years of this study (2017–2019). These values also align with prior reports of influenza sentinel surveillance prevalence across Africa: Niger 6% [9], Uganda 13% [10], Kenya 11% [11], and Nigeria 8% [12]. A large proportion of respiratory disease is not accounted for by influenza virus and these data show the need for broader testing panels, including NIRVs, to identify the likely etiology of respiratory illness [13]. Several lower and middle-income countries have extended the testing algorithms of their influenza surveillance platforms to include RSV, rhinoviruses, adenoviruses, PIVs, and hMPV [14–19]. These studies show that expanding respiratory surveillance by strengthening and building upon an influenza platform is possible, efficient, and practical. In our study, the use of a broader panel of respiratory pathogens increased the number of specimens with a pathogen identified to 61.8% using the CDC singleplex assays and 91.5% using the FTD-33 kit.

Our data indicate that NIRVs are a major cause of respiratory infections in Tanzania, with at least one virus being detected in 62% of cases. The viruses identified most often were RSV, RV and AdV, and they were more common in the younger age groups (particularly <1 year of age). The predominance of these viruses as etiologies for ILI/SARI is a common finding across studies from Africa and worldwide [15, 19, 20]. Additionally, our study shows that odds of hospitalization (SARI vs. ILI) varied by age, sex, location, and year, with hospitalizations being more common among young children (1–<5 years old) and outpatient illness occurring more often in the older age groups (>15 years old). Understanding the age distribution of respiratory viral infections and age-associated care-seeking behavior for severe illness may help guide targeted preventive measures.

Respiratory viruses are present in Tanzania throughout the year; however, using the number of specimens tested as a marker for respiratory infections, the greatest number of ILI/SARI cases were reported from December through April, falling between the two rainy seasons occurring in Tanzania from October–December and March–May. Prior studies have suggested that rainfall might increase exposure to crowded conditions, thus leading to increased risk and incidence of respiratory infections [21]. Influenza A and B viruses and the PIVs showed evidence of seasonality with peaks aligning to the described rainy seasons; this bimodal peak pattern for influenza has previously been described in Tanzania from 2011–2016 [22]. In contrast, 6 years of influenza virus surveillance data in Kenya, the country bordering Tanzania in the north, did not support distinct seasonality for influenza activity in most years [11]; additional data and analyses are needed to understand influenza seasonality in East Africa [23]. RSV also demonstrated evidence of seasonality with higher incidence during the first half of the year, like results from other equatorial countries demonstrating greater viral circulation during their rainy seasons [24]. The pattern for hMPV was similar, consistent with global trends indicating that hMPV often coincides with or follows the peak of RSV activity [25] and also aligning with the distribution previously described for hMPV in Coastal Kenya [26]. Rhinovirus and adenovirus, however, demonstrated no significant seasonality, in concurrence with existing literature [27, 28]. Ultimately, there is considerable controversy around factors contributing to infectious disease seasonality and a consistent explanation remains elusive [29]; continued surveillance in Tanzania may provide additional country-specific insight. Understanding respiratory virus seasonality can allow public health authorities to focus limited resources more efficiently for disease prevention and control at optimum times during the year [30].

This study was subject to several limitations. It was a retrospective analysis and we encountered incomplete data reporting at all sites. Symptom data was only available for the first 2 years and less than 40% of all cases from 2017–2019 reported complete symptom data, making it difficult to analyze symptoms by age or etiology. Analysis of seasonality was limited by the short time frame of data collection (3 years) and specimens were not submitted uniformly throughout the year, with more cases during December–April. Additionally, characteristics of patients presenting to sentinel sites (predominantly regional and district-level hospitals) may not be representative of all patients with ILI/SARI in Tanzania and may not be generalizable to other settings. Particularly, young children represented a large number of ILI/SARI patients (children <1y represented 18.2% of ILI patients and 39.3% of SARI patients; children 1–5y represented 27.2% of ILI patients and 40.4% of SARI patients), which may have been due to greater care-seeking by caretakers of young children. Overrepresentation of these age groups could have biased results regarding etiologies detected and risk of hospitalization. Finally, severe illness may be overrepresented among patients seeking care, and etiologies of ILI/SARI may differ from etiologies of milder, afebrile respiratory illness not captured via sentinel surveillance.

A robust surveillance system can be used to monitor for unusual pathogens, a point made all too clear with the emergence and global spread of the highly infectious respiratory pathogen severe acute respiratory syndrome coronavirus 2 (SARS-CoV-2). Data is not easily extrapolated from other countries as setting-specific estimates may be influenced by study population, the catchment area, case definitions and inclusion criteria, pathogens targeted, and laboratory techniques used to identify the pathogens. In response to the COVID-19 pandemic, influenza surveillance in Tanzania plans to extend testing of the NIRV panel to include SARS-CoV-2. It is vital that we optimize the use of resource and personnel investments to diagnose and surveil for high-burden and emerging pathogens. Lessons learned from the 2017–2019 expansion were that data validation methods need to be established and a strong commitment from both administrative and clinical staff participating in surveillance activities is needed to ensure that all relevant information is collected accurately and completely at each sentinel site. If surveillance data is not being reviewed and analyzed regularly, it cannot be shared forward to decision makers or fed back to those providing the data or other interested parties.

In summary, expanding an existing influenza platform in Tanzania to include additional respiratory pathogens provided valuable insight into the etiology, incidence, severity, and geographic/temporal patterns of respiratory illness. Such pan-respiratory disease surveillance strategies can be used to inform evidence-based interventions to prevent and control illnesses across a range of respiratory pathogens, including coinfections with multiple respiratory pathogens. Strengthening and adapting surveillance systems in the face of emerging respiratory pathogens remains a global health priority; public health authorities and policymakers should continue to assess and adapt programs to provide data for action.

## Supporting information

S1 Text Permission to publish content under CC-BY license for MapChart. (PDF)

S2 Text Inclusivity in global research checklist. (DOCX)

S1 Table Comparison of patient characteristics and pathogen identification for specimens tested using CDC singleplex assays and Fast-Track Diagnostic multiplex kit—Tanzania, 2018–2019. (DOCX)

S2 Table Viral co-detections among ILI/SARI cases—Tanzania, 2017–2019. (DOCX)

S3 Table Viral respiratory pathogens identified, by age group and case classification, among ILI/SARI cases with a single pathogen detected—Tanzania, 2017–2019. (DOCX)

## References

[1] Mmbaga VM, Mwasekaga MJ, Mmbuji P, Matonya M, Mwafulango A, Moshi S, et al. Results from the first 30 months of national sentinel surveillance for influenza in Tanzania, 2008–2010. J Infect Dis. 2012;206 Suppl 1:S80–6. doi: 10.1093/infdis/jis540 23169977

[2] Kishamawe C, Rumisha SF, Mremi IR, Bwana VM, Chiduo MG, Massawe IS, et al. Trends, patterns and causes of respiratory disease mortality among inpatients in Tanzania, 2006–2015. Trop Med Int Health. 2019;24(1):91–100. doi: 10.1111/tmi.13165 30303586

[3] Radin JM, Katz MA, Tempia S, Talla Nzussouo N, Davis R, Duque J, et al. Influenza surveillance in 15 countries in Africa, 2006–2010. J Infect Dis. 2012;206 Suppl 1:S14–21. doi: 10.1093/infdis/jis606 23169960

[4] Tadesse M, Mengesha M, Tayachew A, Belay D, Hassen A, Woyessa AB, et al. Burden and seasonality of medically attended influenza like illness (ILI) in Ethiopia, 2012 to 2017. BMC Infect Dis. 2020;20(1):148. doi: 10.1186/s12879-020-4827-0 32070275 PMC7029599

[5] Yazidi R, Aissi W, Bouguerra H, Nouira M, Kharroubi G, Maazaoui L, et al. Evaluation of the influenza-like illness surveillance system in Tunisia, 2012–2015. BMC Public Health. 2019;19(1):694. doi: 10.1186/s12889-019-7035-3 31170955 PMC6555026

[6] Babakazo P, Kabamba-Tshilobo J, Wemakoy EO, Lubula L, Manya LK, Ilunga BK, et al. Evaluation of the influenza sentinel surveillance system in the Democratic Republic of Congo, 2012–2015. BMC Public Health. 2019;19(1):1652. doi: 10.1186/s12889-019-8008-2 31823763 PMC6902419

[7] Sanou AM, Wandaogo SCM, Poda A, Tamini L, Kyere AE, Sagna T, et al. Epidemiology and molecular characterization of influenza viruses in Burkina Faso, sub-Saharan Africa. Influenza Other Respir Viruses. 2018;12(4):490–6. doi: 10.1111/irv.12539 29350841 PMC6005621

[8] World Health Organization. Global epidemiological surveillance standards for influenza 2013 [Available from: https://www.who.int/publications/i/item/9789241506601].

[9] Maïnassara HB, Lagare A, Tempia S, Sidiki A, Issaka B, Abdou Sidikou B, et al. Influenza Sentinel Surveillance among Patients with Influenza-Like-Illness and Severe Acute Respiratory Illness within the Framework of the National Reference Laboratory, Niger, 2009–2013. PLoS One. 2015;10(7):e0133178. doi: 10.1371/journal.pone.0133178 26230666 PMC4521880

[10] Lutwama JJ, Bakamutumaho B, Kayiwa JT, Chiiza R, Namagambo B, Katz MA, et al. Clinic- and hospital-based sentinel influenza surveillance, Uganda 2007–2010. J Infect Dis. 2012;206 Suppl 1:S87–93. doi: 10.1093/infdis/jis578 23169978

[11] Katz MA, Muthoka P, Emukule GO, Kalani R, Njuguna H, Waiboci LW, et al. Results from the first six years of national sentinel surveillance for influenza in Kenya, July 2007-June 2013. PLoS One. 2014;9(6):e98615. doi: 10.1371/journal.pone.0098615 24955962 PMC4067481

[12] Dalhatu IT, Medina-Marino A, Olsen SJ, Hwang I, Gubio AB, Ekanem EE, et al. Influenza viruses in Nigeria, 2009–2010: results from the first 17 months of a national influenza sentinel surveillance system. J Infect Dis. 2012;206 Suppl 1:S121–8. doi: 10.1093/infdis/jis584 23169957

[13] Tang JW, Lam TT, Zaraket H, Lipkin WI, Drews SJ, Hatchette TF, et al. Global epidemiology of non-influenza RNA respiratory viruses: data gaps and a growing need for surveillance. Lancet Infect Dis. 2017;17(10):e320–e6. doi: 10.1016/S1473-3099(17)30238-4 28457597 PMC7164797

[14] Chittaganpitch M, Waicharoen S, Yingyong T, Praphasiri P, Sangkitporn S, Olsen SJ, et al. Viral etiologies of influenza-like illness and severe acute respiratory infections in Thailand. Influenza Other Respir Viruses. 2018;12(4):482–9. doi: 10.1111/irv.12554 29518269 PMC6005612

[15] Alroy KA, Do TT, Tran PD, Dang TQ, Vu LN, Le NTH, et al. Expanding severe acute respiratory infection (SARI) surveillance beyond influenza: The process and data from 1 year of implementation in Vietnam. Influenza Other Respir Viruses. 2018;12(5):632–42. 29754431 10.1111/irv.12571 PMC6086843

[16] Timmermans A, Melendrez MC, Se Y, Chuang I, Samon N, Uthaimongkol N, et al. Human Sentinel Surveillance of Influenza and Other Respiratory Viral Pathogens in Border Areas of Western Cambodia. PLoS One. 2016;11(3):e0152529. doi: 10.1371/journal.pone.0152529 27028323 PMC4814059

[17] Grunberg M, Sno R, Adhin MR. Epidemiology of respiratory viruses in patients with severe acute respiratory infections and influenza-like illness in Suriname. Influenza Other Respir Viruses. 2021;15(1):72–80. doi: 10.1111/irv.12791 32881286 PMC7767960

[18] Hatem A, Mohamed S, Abu Elhassan UE, Ismael EAM, Rizk MS, El-Kholy A, et al. Clinical characteristics and outcomes of patients with severe acute respiratory infections (SARI): results from the Egyptian surveillance study 2010–2014. Multidiscip Respir Med. 2019;14:11. doi: 10.1186/s40248-019-0174-7 30976418 PMC6442424

[19] Kadjo HA, Ekaza E, Coulibaly D, Kouassi DP, Nzussouo NT, Kouakou B, et al. Sentinel surveillance for influenza and other respiratory viruses in Côte d’Ivoire, 2003–2010. Influenza Other Respir Viruses. 2013;7(3):296–303. 22863403 10.1111/j.1750-2659.2012.00389.x PMC5779848

[20] Ahmed JA, Katz MA, Auko E, Njenga MK, Weinberg M, Kapella BK, et al. Epidemiology of respiratory viral infections in two long-term refugee camps in Kenya, 2007–2010. BMC Infect Dis. 2012;12:7. doi: 10.1186/1471-2334-12-7 22251705 PMC3398263

[21] Murray EL, Klein M, Brondi L, McGowan JE Jr., van Mels C, Brooks WA, et al. Rainfall, household crowding, and acute respiratory infections in the tropics. Epidemiol Infect. 2012;140(1):78–86. doi: 10.1017/S0950268811000252 21371367

[22] Newman LP, Bhat N, Fleming JA, Neuzil KM. Global influenza seasonality to inform country-level vaccine programs: An analysis of WHO FluNet influenza surveillance data between 2011 and 2016. PLoS One. 2018;13(2):e0193263. doi: 10.1371/journal.pone.0193263 29466459 PMC5821378

[23] Hirve S, Newman LP, Paget J, Azziz-Baumgartner E, Fitzner J, Bhat N, et al. Influenza Seasonality in the Tropics and Subtropics—When to Vaccinate? PLoS One. 2016;11(4):e0153003. doi: 10.1371/journal.pone.0153003 27119988 PMC4847850

[24] Obando-Pacheco P, Justicia-Grande AJ, Rivero-Calle I, Rodríguez-Tenreiro C, Sly P, Ramilo O, et al. Respiratory Syncytial Virus Seasonality: A Global Overview. J Infect Dis. 2018;217(9):1356–64. doi: 10.1093/infdis/jiy056 29390105

[25] Kahn JS. Epidemiology of human metapneumovirus. Clin Microbiol Rev. 2006;19(3):546–57. doi: 10.1128/CMR.00014-06 16847085 PMC1539100

[26] Owor BE, Masankwa GN, Mwango LC, Njeru RW, Agoti CN, Nokes DJ. Human metapneumovirus epidemiological and evolutionary patterns in Coastal Kenya, 2007–11. BMC Infect Dis. 2016;16:301. doi: 10.1186/s12879-016-1605-0 27316548 PMC4912817

[27] Horton KC, Dueger EL, Kandeel A, Abdallat M, El-Kholy A, Al-Awaidy S, et al. Viral etiology, seasonality and severity of hospitalized patients with severe acute respiratory infections in the Eastern Mediterranean Region, 2007–2014. PLoS One. 2017;12(7):e0180954. doi: 10.1371/journal.pone.0180954 28704440 PMC5509236

[28] Audi A, AlIbrahim M, Kaddoura M, Hijazi G, Yassine HM, Zaraket H. Seasonality of Respiratory Viral Infections: Will COVID-19 Follow Suit? Front Public Health. 2020;8:567184. doi: 10.3389/fpubh.2020.567184 33042956 PMC7522168

[29] Dowell SF. Seasonality—still confusing. Epidemiol Infect. 2012;140(1):87–90. doi: 10.1017/S0950268811001695 21906417

[30] Azziz Baumgartner E, Dao CN, Nasreen S, Bhuiyan MU, Mah EMS, Al Mamun A, et al. Seasonality, timing, and climate drivers of influenza activity worldwide. J Infect Dis. 2012;206(6):838–46. doi: 10.1093/infdis/jis467 22829641

