# Supplementary material for: Etiologies of influenza-like illness and severe acute respiratory infections in Tanzania, 2017–2019
Source: PLOS Glob Public Health. 2023 Feb 9;3(2):e0000906. doi: 10.1371/journal.pgph.0000906 (PMC10021583; doi:10.1371/journal.pgph.0000906)
Supplement: S1 Text — (PDF) [file pgph.0000906.s001.pdf]

Request for Permission to Publish Content under CC-BY License

Dear Rights Holder or Representative,

I have submitted a paper for publication in a PLOS journal, and wish to include the content listed below in the paper. I'm hereby requesting your (or your company's or institution's) permission to include the content in my paper. Please note that all PLOS journals are published under a Creative Commons Attribution License (CC BY), which allows for unrestricted use and distribution, even commercial, as long as attribution is given to the creator or rights holder of the content. See https://creativecommons.org/licenses/by/4.0/.

To grant me permission to use the content in my PLOS paper, please fill in the information below and then scan the completed form and send it to me at my email address.

Thank you.

My name:

Radhika Gharpure, Epidemiologist, US CDC

My email address:

krr4@cdc.gov

Description of the content which I'm seeking permission to use (citation and/or title, and pasted screen shot, if applicable):

Figure: "Influenza -like illness (ILI) and severe acute respiratory infection (SARI) sentinel surveillance sites in Tanzania, by region and geographic zone."
The map shows the 21 regions (official administrative areas) and 7 geographic zones of mainland Tanzania. Map created with MapChart (openly available source): https://www.mapchart.net/.

Link to the Content:

mapchart.net

* * *

On behalf of myself or the rights holder, I hereby grant the permission sought herein.

Signature of Party Granting Permission:

Date:

28 July 2022

Printed Name and Title:

Minas Giannekas, owner and developer of the MapChart website (https://mapchart.net)
