# Supplementary material for: Etiologies of influenza-like illness and severe acute respiratory infections in Tanzania, 2017–2019
Source: PLOS Glob Public Health. 2023 Feb 9;3(2):e0000906. doi: 10.1371/journal.pgph.0000906 (PMC10021583; doi:10.1371/journal.pgph.0000906)
Supplement: S1 Table — (DOCX) [file pgph.0000906.s003.docx]

**S1 Table: Comparison of patient characteristics and pathogen identification for specimens tested using CDC singleplex assays and Fast-Track Diagnostic multiplex kit — Tanzania, 2018–2019^1^**

|  | **2018** | | | **2019** | | |
| --- | --- | --- | --- | --- | --- | --- |
|  | **CDC**  ***n (%)*** | **FTD-33**  ***n (%)*** | **p-value^2^** | **CDC**  ***n (%)*** | **FTD-33**  ***n (%)*** | **p-value^2^** |
| **Patient demographics** |  |  |  |  |  |  |
| Sex |  |  |  |  |  |  |
| Male | 420 (50.2) | 81 (59.1) | -- | 621 (53.7) | 82 (69.5) | -- |
| Female | 416 (49.8) | 56 (40.9) | 0.05 | 535 (46.3) | 36 (30.5) | 0.001 |
| Age group (years) |  |  |  |  |  |  |
| <1 | 249 (29.8) | 39 (28.5) | -- | 388 (33.6) | 39 (33.1) | -- |
| 1–<5 | 298 (35.7) | 59 (43.1) | -- | 383 (33.1) | 42 (35.6) | -- |
| 5–<18 | 71 (8.5) | 15 (11.0) | -- | 89 (7.7) | 5 (4.2) | -- |
| 18–<65 | 187 (22.4) | 20 (14.6) | -- | 256 (22.2) | 31 (26.3) | -- |
| 65+ | 31 (3.7) | 4 (2.9) | 0.19 | 40 (3.5) | 1 (0.9) | 0.29 |
| **Case classification** |  |  |  |  |  |  |
| ILI | 272 (32.5) | 39 (28.5) | -- | 430 (37.2) | 60 (50.9) | -- |
| SARI | 564 (67.5) | 98 (71.5) | 0.34 | 726 (62.8) | 58 (49.2) | 0.004 |
| **Pathogens identified** |  |  |  |  |  |  |
| RSV | 191 (22.9) | 25 (18.3) | 0.23 | 309 (26.7) | 43 (36.4) | 0.02 |
| Rhinovirus | 179 (21.4) | 21 (15.3) | 0.10 | 243 (21.0) | 25 (21.2) | 0.97 |
| Adenovirus | 96 (11.5) | 9 (6.6) | 0.09 | 185 (16.0) | 27 (22.9) | 0.06 |
| hMPV | 37 (4.4) | 2 (1.5) | 0.10 | 89 (7.7) | 8 (6.8) | 0.72 |
| Influenza A | 36 (4.3) | 7 (5.1) | 0.18 | 49 (4.2) | 7 (5.9) | 0.58 |
| PIV3 | 35 (4.2) | 4 (2.9) | 0.48 | 55 (4.8) | 6 (5.1) | 0.87 |
| Influenza B | 50 (6.0) | 5 (3.7) | 0.27 | 14 (1.2) | 0 (0) | 0.23 |
| PIV1 | 24 (2.9) | 2 (1.5) | 0.34 | 32 (2.8) | 2 (1.7) | 0.49 |
| PIV2 | 8 (1.0) | 4 (2.9) | 0.05 | 16 (1.4) | 2 (1.7) | 0.79 |

Abbreviations: CDC, U.S. Centers for Disease Control singleplex assays; FTD-33, Fast-Track Diagnostics multiplex kit; ILI, influenza-like illness; SARI, severe acute respiratory illness; RSV, respiratory syncytial virus; hMPV, human metapneumovirus; PIV, parainfluenza virus (1, 2, and 3).

^1^No specimens were tested using the FTD-33 assay in 2017.

^2^p-value from Pearson’s chi-squared test comparing CDC and FTD-33 assays. Proportion of specimens testing positive vs. negative were compared for each pathogen individually.
