# Supplementary material for: Etiologies of influenza-like illness and severe acute respiratory infections in Tanzania, 2017–2019
Source: PLOS Glob Public Health. 2023 Feb 9;3(2):e0000906. doi: 10.1371/journal.pgph.0000906 (PMC10021583; doi:10.1371/journal.pgph.0000906)
Supplement: S3 Table — (DOCX) [file pgph.0000906.s005.docx]

**S3 Table: Viral respiratory pathogens identified, by age group and case classification, among ILI/SARI cases with a single pathogen detected — Tanzania, 2017–2019**

|  |  | **ILI** | | | | |  | | **SARI** | | | | |  | |  |
| --- | --- | --- | --- | --- | --- | --- | --- | --- | --- | --- | --- | --- | --- | --- | --- | --- |
|  |  | **Age group (years)**  **n (%)** | | | | |  | | **Age group (years)**  **n (%)** | | | | |  | |  |
| **Pathogen** | **Total** | **<1** | **1–<5** | **5–<18** | **18–<65** | **≥65** | | **Total** | **<1** | **1–<5** | **5–<18** | **18–<65** | **≥65** | | **Total** | **p-value^2^** |
| RSV | 409 | 33 (27.1) | 43 (35.3) | 14 (11.5) | 30 (24.6) | 2 (1.6) | | 122 | 154 (53.7) | 105 (36.6) | 14 (4.9) | 11  (3.8) | 3 (1.1) | | 287 | <0.0001 |
| Rhinovirus | 388 | 27 (19.3) | 36 (25.7) | 16 (11.4) | 55 (39.3) | 6 (4.3) | | 140 | 96 (38.7) | 98 (39.5) | 19 (7.7) | 29 (11.7) | 6 (2.4) | | 248 | <0.0001 |
| Adenovirus | 163 | 12 (23.5) | 19 (37.3) | 4  (7.8) | 14 (27.5) | 2 (3.9) | | 51 | 45 (40.2) | 57 (50.9) | 2  (1.8) | 6  (5.4) | 2 (1.8) | | 112 | 0.0002 |
| Influenza A | 86 | 0  (0.0) | 7 (18.4) | 8 (21.1) | 21 (55.3) | 2 (5.3) | | 38 | 14 (29.2) | 34 (50.0) | 4  (8.3) | 4  (8.3) | 2 (4.2) | | 48 | <0.0001 |
| hMPV | 69 | 4 (19.1) | 4 (19.1) | 4 (19.1) | 8  (38.1) | 1 (4.8) | | 21 | 20 (41.7) | 22 (45.8) | 4  (8.3) | 2  (4.2) | 0  (0.0) | | 48 | 0.0004 |
| PIV3 | 66 | 8 (25.8) | 11 (35.5) | 0  (0.0) | 11 (35.5) | 1 (3.2) | | 31 | 14 (40.0) | 11 (31.4) | 3  (8.6) | 5  (14.3) | 2 (5.7) | | 35 | 0.1357 |
| Influenza B | 48 | 0  (0.0) | 3 (11.1) | 10 (37.0) | 12 (44.4) | 2 (7.4) | | 27 | 4 (19.1) | 9 (42.9) | 3 (14.3) | 4  (19.1) | 1 (4.8) | | 21 | 0.0057 |
| PIV1 | 26 | 1 (14.3) | 1 (14.3) | 1 (14.3) | 3  (42.9) | 1 (14.3) | | 7 | 5 (26.3) | 9 (47.4) | 1  (5.3) | 3  (15.8) | 1 (5.3) | | 19 | 0.3445 |
| PIV2 | 11 | 0  (0.0) | 1 (20.0) | 1 (20.0) | 3  (60.0) | 0  (0.0) | | 5 | 2 (33.3) | 3 (50.0) | 0  (0.0) | 1  (16.7) | 0  (0.0) | | 6 | 0.1755 |
| No virus identified^3^ | 1044 | 54 (15.9) | 72 (21.2) | 43 (12.7) | 158 (46.5) | 13 (3.8) | | 340 | 222 (31.5) | 276 (39.2) | 48 (6.8) | 121 (17.2) | 37 (5.3) | | 704 | <0.0001 |

Abbreviations: ILI, influenza-like illness; SARI, severe acute respiratory illness; RSV, respiratory syncytial virus; hMPV, human metapneumovirus; PIV, parainfluenza virus (1, 2, and 3); CDC, U.S. Centers for Disease Control singleplex assays; FTD-33, Fast-Track Diagnostics multiplex kit.

^1^Patients with multiple viruses detected (n=420) were not included in this analysis.

^2^p-value for chi-squared test comparing age distributions among ILI and SARI cases for each viral pathogen.

^3^Among nine viruses included on both CDC and FTD-33 diagnostic assays; additional viral pathogens were tested using the FTD-33 kit on a subset of specimens and were not included in this analysis.
